# Genetic Variants in *EPAS1* Contribute to Adaptation to High-Altitude Hypoxia in Sherpas

**DOI:** 10.1371/journal.pone.0050566

**Published:** 2012-12-05

**Authors:** Masayuki Hanaoka, Yunden Droma, Buddha Basnyat, Michiko Ito, Nobumitsu Kobayashi, Yoshihiko Katsuyama, Keishi Kubo, Masao Ota

**Affiliations:** 1 First Department of Medicine, Shinshu University School of Medicine, Matsumoto, Japan; 2 Nepal International Clinic, Katmandu, Nepal; 3 Department of Pharmacy, Shinshu University Hospital, Matsumoto, Japan; 4 Department of Legal Medicine, Shinshu University School of Medicine, Matsumoto, Japan; University of Florence, Italy

## Abstract

Sherpas comprise a population of Tibetan ancestry in the Himalayan region that is renowned for its mountaineering prowess. The very small amount of available genetic information for Sherpas is insufficient to explain their physiological ability to adapt to high-altitude hypoxia. Recent genetic evidence has indicated that natural selection on the *endothelial PAS domain protein 1 (EPAS1*) gene was occurred in the Tibetan population during their occupation in the Tibetan Plateau for millennia. Tibetan-specific variations in *EPAS1* may regulate the physiological responses to high-altitude hypoxia via a hypoxia-inducible transcription factor pathway. We examined three significant tag single-nucleotide polymorphisms (SNPs, rs13419896, rs4953354, and rs4953388) in the *EPAS1* gene in Sherpas, and compared these variants with Tibetan highlanders on the Tibetan Plateau as well as with non-Sherpa lowlanders. We found that Sherpas and Tibetans on the Tibetan Plateau exhibit similar patterns in three *EPAS1* significant tag SNPs, but these patterns are the reverse of those in non-Sherpa lowlanders. The three SNPs were in strong linkage in Sherpas, but in weak linkage in non-Sherpas. Importantly, the haplotype structured by the Sherpa-dominant alleles was present in Sherpas but rarely present in non-Sherpas. Surprisingly, the average level of serum erythropoietin in Sherpas at 3440 m was equal to that in non-Sherpas at 1300 m, indicating a resistant response of erythropoietin to high-altitude hypoxia in Sherpas. These observations strongly suggest that *EPAS1* is under selection for adaptation to the high-altitude life of Tibetan populations, including Sherpas. Understanding of the mechanism of hypoxia tolerance in Tibetans is expected to provide lights to the therapeutic solutions of some hypoxia-related human diseases, such as cardiovascular disease and cancer.

## Introduction

Sherpas are originally Tibetans who emigrated from eastern Tibet to the Everest region of Nepal 500 years ago in order to be close to the mountain they hold sacred, Mt. Everest according to the evidences of the history of Sherpas and linguistics [1], [2]. Nowadays, most Sherpas reside at elevations above 3000 m in the Himalayan region in Nepal. Their mountaineering prowess and power of endurance has brought them prominence in adventure mountaineering and has attracted the interest of anthropologists, physiologists, mountain medicine researchers, and sports scientists. Investigations have employed morphological [3], physiological [4]–[6], and genetic [7]–[9] methods to describe Sherpa adaptations to high altitude. Yet, the present available genetic evidences are insufficient to explain the Sherpas’ powerful adaptation to high-altitude hypoxia at genetic level.

For decades, studies have addressed the mechanisms of genetic adaptation to high-altitude hypoxia that have allowed people of Tibetan ethnicity to permanently inhabit the Tibetan Plateau at elevations of 3500–4500 m for ∼25,000 years [10]–[17]. How do the Tibetan indigenous highlanders negotiate the unique stresses of hypobaric hypoxia, surviving and reproducing on the Tibetan Plateau where most lowland humans become life-threateningly ill and must descend? Hypoxia is a key factor involving most of the major biological pathways that contribute to human pathogeneses of the cardiovascular system, the respiratory system, neurophysiology, oncology, transplantation, and infectious diseases, and it has been prospected that understanding the mechanism of hypoxia tolerance in Tibetans may provide lights to the therapeutic solutions of these diseases.

Analyses of genomes in the high-altitude populations may reveal concerned signaling pathways that may account for high-altitude adaptation. The advanced genome-wide scanning techniques were applied to detect signatures of natural selection and identify genes and genetic variants that contribute to human adaptation to altitude in Tibetan [12]–[17] and Andean [18] high-altitude populations. The *EPAS1* (endothelial PAS domain protein 1), *EGLN1* (early growth response 1) and *PPARA* (peroxisome proliferator activated receptor alpha) genes were the most notably candidate genes that have been identified in the roles of evolutionary adaptation to high altitude in Tibetans, probably by some unknown molecular pathways that delicately control erythropoietic response to hypoxia [12], [13]. Recent genetic evidence has indicated that natural selection has acted on the *EPAS1* gene in the Tibetan population during their occupation of the Tibetan Plateau [12]–[17]. Human *EPAS1* (also known as hypoxia-inducible factor-2, *HIF-2*) encodes the oxygen-sensitive subunit of the HIF-2 transcription factor and plays an important role in regulating erythropoiesis [19]. Tibetan-specific allelic variations in *EPAS1* regulate the physiological responses to high-altitude hypoxia through the HIF signaling pathway to maintain the hemoglobin (Hb) levels of Tibetan highlanders at near-sea-level values [12], [20]. The lower-than-expected Hb concentration at high altitude is one of the major phenotypes in high-altitude adaptation in Tibetans living on the Tibetan Plateau [20]–[22]. The purpose of the current study was to examine three significant Tibetan-specific allelic variations in *EPAS1* in a Sherpa population, providing fresh genetic information on the mechanisms of genetic adaptation to high-altitude hypoxia, given the previous observations [12]–[17] of natural selection acting on *EPAS1* in Tibetans on the Tibetan Plateau.

## Results

**Figure 1:**
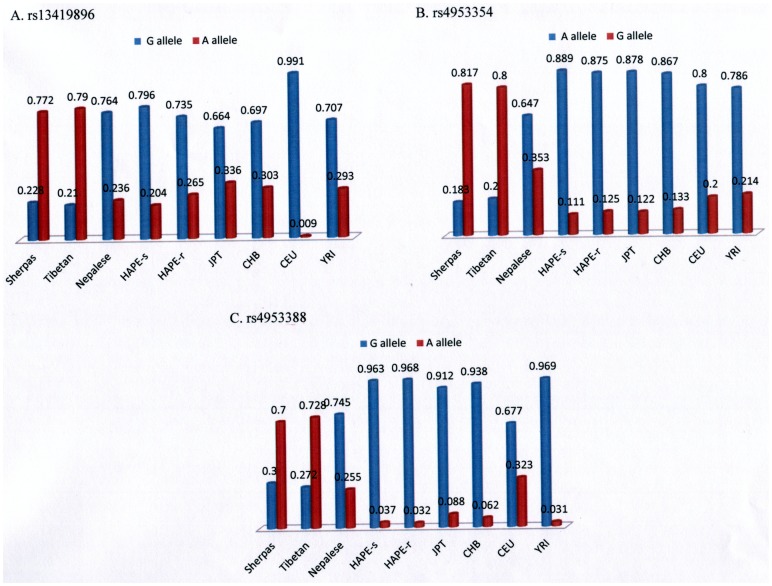
Patterns and frequencies of the three single nucleotide polymorphisms. Patterns of the predominant alleles of rs13419896 (A), rs4953354 (B), and rs4953388 (C) on Chromosome 2 in Sherpas, Tibetans, and other non-Sherpa lowlander populations worldwide. Red, frequency of the Sherpa-dominant allele; blue, frequency of the ancestral allele defined in the NCBI dbSNP database. Abbreviations: CEU, Utah residents with Northern and Western European ancestry; CHB, Han Chinese in Beijing, China; HAPE-r, Japanese high-altitude pulmonary edema-resistant subjects; HAPR-s, Japanese high-altitude pulmonary edema-susceptible subjects; JPT, Japanese subjects in Tokyo, Japan; YRI, Yoruban subjects in Ibadan, Nigeria.

The patterns of the predominant alleles of three tag single nucleotide polymorphisms (SNPs; rs13419896, rs4953354, and rs4953388) in Sherpas were very similar to those in Tibetans on the Tibetan Plateau, but were the reverse of those in non-Sherpa populations including Nepalese subjects, Japanese high-altitude pulmonary edema (HAPE)-susceptible subjects (J-HAPE-s, a group of Japanese individuals that were susceptible to high-altitude pulmonary edema due to genetic mutations [23]–[25]), Japanese HAPE-resistant subjects (J-HAPE-r, a group of Japanese individuals resistant to high-altitude pulmonary edema due to a non-mutated genetic background [23]–[25]), Japanese subjects in Tokyo (JPT), Han Chinese in Beijing (CHB), Utah residents with ancestry from northern and western Europe (CEU), and Yoruba from Ibadan (YRI; Table 1, Figure 1). The major alleles of the three SNPs in Sherpas were distributed in parallel with those in Tibetans (rs13419896/A, rs4953354/G, and rs4953388/A), but divergent from those in non-Sherpa populations (rs13419896/G, rs4953354/A, and rs4953388/G). This observation indicates that the high-altitude populations (Sherpas in the Himalayan region in Nepal and Tibetans on the Tibetan Plateau) and low-altitude populations (JPT, CHB, CEU, YRI) are clearly separated into two genetic clusters by the distributions of the major alleles of *EPAS1* gene.

**Table 1:** Data for rs13419896, rs4953354, and rs4953388 for Sherpas, Tibetans, and other non-Sherpa lowlander populations worldwide.

SNP	Sherpas	Tibetans	Nepalese	J-HAPE-s	J-HAPE-r	JPT*	CHB*	CEU*	YRI*
**rs13419896 (Position: 46556345; Function: intron in ** ***EPAS1*** ** gene)**									
**Genotype (G/A = ancestral allele/derived allele according to NCBI dbSNP database)**									
G/G	0.068	–	0.618	0.611	0.545	0.46	0.467	0.982	0.476
G/A	0.32	–	0.291	0.37	0.379	0.407	0.46	0.018	0.463
A/A	0.612	–	0.091	0.019	0.076	0.133	0.073	0	0.061
**Allele**									
G	0.228	0.210†	0.764	0.796	0.735	0.664	0.697	0.991	0.707
A	0.772	0.790†	0.236	0.204	0.265	0.336	0.303	0.009	0.293
**rs4953354 (Position: 46575388; Function: intron in ** ***EPAS1*** ** gene)**									
**Genotype (A/G)**									
A/A	0.058	–	0.44	0.778	0.75	0.756	0.733	0.692	0.603
A/G	0.25	–	0.413	0.222	0.25	0.244	0.267	0.215	0.365
G/G	0.692	–	0.147	0	0	0	0	0.092	0.032
**Allele**									
A	0.183	0.200†	0.647	0.889	0.875	0.878	0.867	0.8	0.786
G	0.817	0.800†	0.353	0.111	0.125	0.122	0.133	0.2	0.214
**rs4953388 (Position: 46713201; Function: unknown gene downstream of ** ***EPAS1*** ** gene)**									
**Genotype (G/A)**									
G/G	0.085	–	0.536	0.926	0.937	0.823	0.876	0.496	0.939
G/A	0.429	–	0.418	0.074	0.063	0.177	0.124	0.363	0.061
A/A	0.486	–	0.046	0	0	0	0	0.142	0
**Allele**									
G	0.3	0.272‡	0.745	0.963	0.968	0.912	0.938	0.677	0.969
A	0.7	0.728‡	0.255	0.037	0.032	0.088	0.062	0.323	0.031

Abbreviations: CEU, Utah residents with Northern and Western European ancestry; CHB, Han Chinese in Beijing, China; J-HAPE-r, Japanese high-altitude pulmonary edema-resistant subjects; J-HAPR-s, Japanese high-altitude pulmonary edema-susceptible subjects; JPT, Japanese subjects in Tokyo, Japan; YRI, Yoruban subjects in Ibadan, Nigeria.

* Genetic data from HapMap (http://hapmap.ncbi.nlm.nih.gov/index.html.en).

† Genetic data from reference 16.

‡ Genetic data from reference 15.

The genetic distances (*F_ST_*) of the three tag SNPs were considerably large between Sherpas and J-HAPE-r subjects, with *F*_ST_ values of 0.481 for rs4953354 and rs4953388 and of 0.257 for rs13419896 (Table 2). The genetic distances were reasonably separated between Sherpas and non-Sherpa Nepalese, with *F_ST_* values of 0.287 for rs13419896, 0.222 for rs4953354, and 0.198 for rs4953388. However, the genetic distances of the three tag SNPs were relatively near between the two lowland populations of non-Sherpa Nepalese and J-HAPE-r subjects (Table 2). Taken together, these observations indicate that the three *EPAS1* SNPs were distinguishably present in the highlander Sherpas. The *F_ST_* value for Sherpas and Tibetans on the Tibetan Plateau was not calculated because the genotype information for the three tag SNPs in Tibetans on the Tibetan Plateau was unavailable from related studies [12]–[17].

**Table 2:** Pair-wise genetic distances (FST) for Sherpa, non-Sherpa Nepalese, and J-HAPE-r populations for each single nucleotide polymorphism (SNP).

SNPs	Sherpas–Nepalese	Sherpas–J-HAPE-r	Nepalese–J-HAPE-r
rs13419896	0.287	0.257	0.001
rs4953354	0.222	0.481	0.072
rs4953388	0.198	0.481	0.101

Abbreviations: J-HAPE-r, Japanese high-altitude pulmonary edema-resistant subjects.

**Figure 2:**
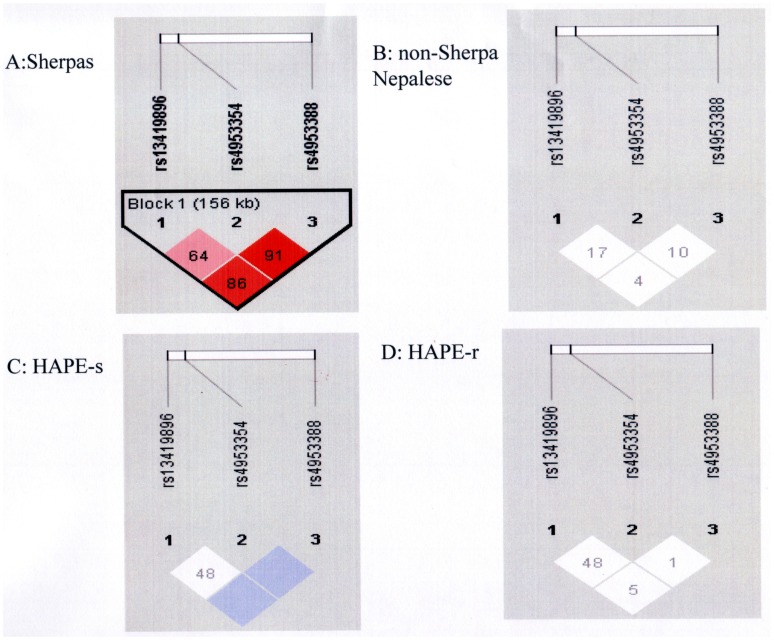
Linkage disequilibrium of the three single nucleotide polymorphisms. Linkage disequilibrium of the three *EPAS1* single nucleotide polymorphisms in Sherpas (A), non-Sherpa Nepalese (B), Japanese high-altitude pulmonary edema-susceptible subjects (HAPE-s; C) and Japanese high-altitude pulmonary edema-resistant subjects (HAPE-r, D). *D’* values appear in the diamonds. Red, D’>0.8 with logarithm of the odds (LOD)≥3; pink, D’<0.8 with LOD≥3; blue, D’>0.8 with LOD<3; white, D’<0.8 with LOD<3.

Pair-wise linkage disequilibrium (LD) analyses were performed for Sherpa, non-Sherpa Nepalese, J-HAPE-s, and J-HAPE-r populations in the present study. The three tag SNPs were highly linked as a block on Chromosome 2 in Sherpas, and the rs4953388 allele was in the leading position of the linkage as indicated by the D’, r^2^ and logarithm of the odds (LOD) in Table 3 and Figure 2. In J-HAPE-s subjects the LD was strong (D’ = 1 for the rs4953388-rs13419896 and rs4953388-rs4953354 pairs); however, the LOD scores were <3, indicating low-confidence D’ values for this group. No linkage of the three tag SNPs was detected in non-Sherpa Nepalese and J-HAPE-r subjects.

**Table 3:** Pair-wise linkage disequilibrium of the three tag SNPs in Sherpa, non-Sherpa Nepalese, J-HAPE-s, and J-HAPE-r populations.*

SNP1	SNP2	D'	r^2^	LOD
***Sherpas***				
rs13419896	rs4953354	0.64	0.323	8.54
rs13419896	rs4953388	0.868	0.529	15.75
rs4953354	rs4953388	0.918	0.458	13.6
***non-Sherpa Nepalese***				
rs13419896	rs4953354	0.179	0.018	0.58
rs13419896	rs4953388	0.043	0.002	0.04
rs4953354	rs4953388	0.105	0.007	0.15
***J-HAPE-s***				
rs13419896	rs4953354	0.487	0.116	1
rs13419896	rs4953388	1	0.01	0.4
rs4953354	rs4953388	1	0.005	0.21
***J-HAPE-r***				
rs13419896	rs4953354	0.482	0.078	0.92
rs13419896	rs4953388	0.053	0	0
rs4953354	rs4953388	0.017	0	0

Abbreviations: LOD, logarithm of the odds; J-HAPE-r, Japanese high-altitude pulmonary edema-resistant subjects; J-HAPE-s, Japanese high-altitude pulmonary edema-susceptible subjects, SNP, single nucleotide polymorphism.

* D’ values, r^2^ values, and LOD were examined with Haploview 3.32.

The frequency of the haplotype of the Sherpa-dominant alleles (**A**/rs13419896-**G**/rs4953354-**A**/rs4953388) was significantly higher in Sherpas (0.684) than in non-Sherpa Nepalese (0.048, P<1.85×10^−22^) and Japanese HAPE subjects (J-HAPE-s: frequency = 0, P<2.26×10^−16^; J-HAPE-r: frequency = 0, P<1.12×10^−18^; Table 4). On the other hand, the haplotype constructed by the dominant alleles in the NCBI dbSNP database (**G**/rs13419896-**A**/rs4953354-**G**/rs4953388) was substantially present in the J-HAPE-s (0.713) and J-HAPE-r (0.660) groups, moderately present in the non-Sherpa Nepalese group (0.381), and marginally present in Sherpas (0.128; P<9.43×10^−14^, 6.75×10^−13^, and 2.16×10^−5^ for Sherpas vs. J-HAPE-s, Sherpas vs. J-HAPE-r, and Sherpas vs. non-Sherpa Nepalese, respectively). Other minor haplotypes (G-G-G, A-A-G, A-G-G, G-G-A, and G-A-A) were present at low frequencies in Sherpa and non-Sherpa populations (Table 4).

**Table 4:** Frequencies of the haplotypes constructed by the three tag single nucleotide polymorphisms in Sherpa, non-Sherpa Nepalese, J-HAPE-s, and J-HAPE-r populations.

Haplotype structures			Populations			
rs13419896(G/A*)	rs4953354 (A/G*)	rs4953388 (G/A*)	Sherpas	Nepalese (P value†)	J-HAPE-s (P value†)	J-HAPE-r (P value†)
A	G	A	0.684	0.048 (1.85×10^−22^)	0 (2.26×10^−16^)	0 (1.12×10^−18^)
G	A	G	0.128	0.381 (2.16×10^−5^)	0.713 (9.43×10^−14^)	0.660 (6.75×10^−13^)
G	G	G	0.079	0.181 (0.0265)	0.046 (0.4331)	0.045(0.3829)
A	A	G	0.048	0.118 (0.0637)	0.139 (0.0438)	0.196 (0.0021)
A	G	G	0.04	0.068 (0.3644)	0.065 (0.48709	0.067 (0.4321)
G	G	A	0.011	0.057 (0.0646)	0 (0.4392)	0 (0.3926)
G	A	A	0	0.142 (6.06×10^−5^)	0.037 (0.04739	0.024 (0.1107)

Abbreviations: J-HAPE-r, Japanese high-altitude pulmonary edema-resistant subjects; J-HAPE-s, Japanese high-altitude pulmonary edema-susceptible subjects.

* Ancestral and derived alleles according to the NCBI dbSNP database.

† Significant differences between the given population and the Sherpa population by Chi square test.

The serum levels of erthyropoietin (EPO) were measured in Sherpas and non-Sherpa Nepalese (fresh serum samples were not available for the other groups). Surprisingly, the average serum EPO level in Sherpas at 3440 m (23.1±11.2 mU/mL) was equivalent to that in non-Sherpa Nepalese at 1300 m (22.5±15.4 (mU/mL), indicating an EPO-mediated resistance to high-altitude hypoxia in Sherpas (Table 5). However, we did not detect any significant association of the genotypes of the three tag SNPs with the serum levels of EPO in Sherpas or in non-Sherpa Nepalese (Table 5).

**Table 5:** Serum levels of erythropoietin by genotype in Sherpas and non-Sherpa Nepalese.*

	Sherpas at 3440 m	Nepalese at 1300 m
**Overall levels**	23.1±11.2 (mU/mL)	22.5±15.4 (mU/mL)
**By genotypes**		
rs13419896 (G/A)		
GG	25.7±11.8	21.6±8.3
GA	20.5±9.7	20.1±8.7
AA	22.7±8.6	17.1±11.2
rs4953354 (A/G)		
AA	25.8±14.9	21.6±10.4
AG	22.8±11.3	20.7±5.9
GG	22.1±8.1	18.2±9.4
rs4953388 (G/A)		
GG	20.2±7.7	19.8±7.7
GA	23.0±9.9	21.1±8.3
AA	22.1±8.7	23.4±11.1

* Data are expressed as means ± standard deviation. P>0.05 for the comparisons of each genotype between Sherpa and non-Sherpa Nepalese subjects, calculated by unpaired Student’s t-test.

## Discussion

This study provides genetic evidence from Sherpas to support the recent observations that natural selection acting on *EPAS1* has contributed to high-altitude adaptation for Tibetans [12]–[17]. The present study also demonstrates that variants in *EPAS1* are essential parts of the fundamental genetic background of Sherpas, who naturally able to survive and reproduce in the Himalayan high-altitude region.

Accumulated information on the genetic background of Tibetans on the Tibetan Plateau has consistently demonstrated natural selection of *EPAS1* in indigenous Tibetans and its decisive roles in adaptation to high-altitude hypoxia by Tibetans [12]–[17], who have lived in high-altitude geographical areas for millennia [26]. The allelic frequencies of the three *EPAS1* SNPs in Sherpas were equivalent to those in Tibetans on the Tibetan Plateau, but divergent from those in non-Sherpa populations worldwide (Table 1). Likewise, the patterns of the predominant SNP alleles in Sherpas were similar to those in Tibetans on the Tibetan Plateau but the reverse of those in non-Sherpa populations (Figure 1). The genetic distances of the three SNPs were quite large between Sherpas and non-Sherpas (Table 2) and between Tibetans on the Tibetan Plateau and non-Tibetans as previously reported [16]. Strong pair-wise LD occurred in these SNPs in Sherpas, covering 56 kb from rs13419896 in intron 1 to rs4953388 downstream of *EPAS1*, but these SNPs were in weak linkage in non-Sherpas. Such LD was also detected in Tibetans on the Tibetan Plateau, but had low linkage in non-Tibetans [12]. The haplotype structured by the Sherpa-dominant alleles (**A**/13419896-**G**/rs4953354-**A**/rs4953388) was present in Sherpas, but only occasionally present in non-Sherpa Nepalese subjects and nonexistent in Japanese subjects. Our data provides further evidence for the proposal that the *EPAS1* variants underlying this haplotype confer a specific advantage in adaptation to high altitude [12]–[17]. The present genetic data in Sherpas provide compelling evidence that *EPAS1* is under natural selection in Tibetans, including Sherpas, under the environmental pressure of high-altitude hypoxia.

The roles of Sherpas in expeditions to the extreme altitudes of the peaks and passes in the Himalayan region are irreplaceable because of their resistance to hypoxia which is believed to be due to a genetic background that provides morphological and physiological adaptations to high altitude [3]–[9], [27]. *EPAS1* encodes the HIF-2α protein, which up-regulates numerous hypoxia-inducible genes (including *EPO*) in the lung and other tissues experiencing hypoxia, even during embryonic development [28]. The up-regulated EPO amplifies the proliferation and differentiation of red blood cell precursors, resulting in an increase of Hb over than the sea-level value. Increased EPO plays a key role in the development of polycythemia [19], one of the clinical characteristics in chronic mountain sickness caused by the progressive failure of homeostatic control of Hb concentration [29]. The rise in Hb concentrations to levels above those found at sea level increases the oxygen content of the blood but does not necessarily increase oxygen delivery to the tissues, eventually resulting in hypoxemia, hypoxic pulmonary hypertension, and high-altitude heart disease [29]. The present genetic evidence together with the findings of sea-level concentration of serum EPO in Sherpas at 3440 m further supported the proposal that the genetic variants in *EPAS1* eventually encode hypoxia-blunted HIF-2α proteins that likely down-regulate *EPO* under hypoxia via unknown molecular pathways [12]. Lower-than-expected hematocrit and Hb values for the given high altitude have been recorded in Tibetans on the Tibetan Plateau and Sherpas [20]–[22], [30], [31]. Sea-level amounts of Hb, hematocrit, and red blood cells are the best physiological requirements for the hemoglobin-oxygen transport system to efficiently carry and deliver oxygen to the tissue, cellular, and molecular levels of the organism [32]. The current finding that the average level of serum EPO in Sherpas at 3440 m was the same as that in non-Sherpas at low altitude (Table 5) evidenced a down-regulated EPO phenotype in Sherpas at high altitude. Of course, the contributions of *EPAS1* to high-altitude adaptation are not limited to the hemoglobin-oxygen transport system, since *EPAS1* was shown to influence the aerobic-anaerobic condition of elite endurance athletes as well [33]. *EPAS1* is a sensor capable of integrating cardiovascular function, energetic demand, muscular activity, and oxygen availability into a physiological adaptation [34], [35] that occurs in Sherpas at high altitude [3]–[9].

*EPAS1* has 16 exons extending over 90 kb of genomic DNA, with a large first intron (50 kb) [36]. Most of the SNPs (*F*_ST_>0.3) that were highly differentiated between Tibetans on the Tibetan Plateau and non-Tibetan CHB occur within the introns of *EPAS1* according to previous studies [16], [17]. The exonic SNPs were not included in the current study due to a lack of SNP information in the exons of *EPAS1* in Tibetans. It is urgent to carry out advanced studies to find the functional SNPs in the exons of the *EPAS1* and elucidate their roles in the adaptation to high altitude in Tibetan ethnicity.

In conclusion, the patterns of the predominant-alleles of the three tag *EPAS1* SNPs in Sherpas paralleled the Tibetan-specific genetic variants, strongly suggesting th*EPAS1* is under selection in people of Tibetan ethnicity. The Sherpa-dominant *EPAS1* haplotype may establish a fundamental genetic background for hypoxia-tolerant oxygen sensing, resulting in an efficient hemoglobin-oxygen transport system in Sherpas at high altitude.

## Materials and Methods

### Ethics Statement

The current study was approved by the Ethics Committee of Shinshu University (Matsumoto, Japan) and the Nepal Health Research Council (Kathmandu, Nepal). The protocol of the investigation was in accordance with the principles outlined in the Declaration of Helsinki of the World Medical Association [37] and approved by both the Ethics Committee of Shinshu University and the Nepal Health Research Council. The protocol was explained to each Sherpa and non-Sherpa Nepalese individually and an informed consent written in Nepali was obtained by either signature or fingerprint if the subject could not write.

### Populations

Blood samples were obtained from 105 Sherpas residing in Namche Bazaar (3440 m above sea level, the largest Sherpa village in the Solu-Khumbu region of Nepal) and nearby villages (Khumjung, Kunde Thame, Forte, Pangboche). Sherpas were randomly recruited in the villages. Membership in the Sherpa clan was determined on the basis of self-report with their ethnic “Sherpa” surname and confirmed by a senior native Sherpa who was a coordinator for our fieldwork. All enrolled Sherpas were permanent residents in these villages without a history of intermarriage with other ethnic groups. Thirty-three (31.4%) Sherpa men and women were frequently employed as trekking guides and porters by expedition teams; 13 Sherpa men had climbed mountains over 8000 m and six of them had reached the summit of Mt. Everest (8848 m) several times. The average height of the mountains they climbed was (5701.4±119.1 m). The average values for percutaneous oxygen saturation and heart rate were 93.3±0.2% and 80.7±1.1 beats per minute, respectively, at a high altitude of 3440 m.

In contrast to Sherpas living at high altitude, non-Sherpa Nepalese in Kathmandu (1330 m) reside at low altitude in Nepal. The two populations are not only geographically close, but they have also been associated politically, economically, and culturally for the last five centuries. Therefore, assuming maladaptation to high altitude, the non-Sherpa citizens of Kathmandu were chosen as a reference population for comparison with Sherpas at high altitude. Blood samples were obtained from 111 non-Sherpa healthy unrelated Nepalese (university students and hospital staff in the city, farmers and housewife in the countryside) in Kathmandu (1330 m). DNA samples from 54 J-HAPE-s and 66 J-HAPE-r subjects were collected during our previous case-control association studies of HAPE [23], [24] and were utilized in the current study. The J-HAPE-s and J-HAPE-r groups are thought to be genetically distinguished with respect to their biological sensitivity to high-altitude hypoxia [23]–[25]. The relatively close genetic distance between Tibetans on the Tibetan Plateau and Japanese (*F_ST_* = 0.017) based on whole-genome data [15] theoretically allowed a reasonable genetic comparison between Sherpa and Japanese subjects.

Allelic information for the three *EPAS1* SNPs in Tibetans permanently residing on the Tibetan Plateau was taken from previous reports [12], [15], [16] for a genetic comparison between Sherpas in Nepal and Tibetans on the Tibetan Plateau.

In order to determine the degree of divergence of the *EPAS1* tag SNPs between Sherpa and non-Sherpa populations worldwide, data for these SNPs in the four HapMap populations (JPT, CHB, CEU, and YRI) were obtained as well [38].

### Selection of EPAS1 Tag SNPs

SNP rs13419896 (G (ancestral allele)/A (derived allele), according to the NCBI dbSNP database) is located at position 46556345 on Chromosome 2, within intron 1 of *EPAS1*. A single causal variant model identified that the SNP rs13419896 was the most significant SNP that correlated with a lower-than-expected Hb concentration for an altitude of 4300 m in a group of Tibetans on the Tibetan Plateau, among 32 significantly associated SNPs [12]. SNP rs4953354 (A/G) occurs at position 46575388 on Chromosome 2, within intron 2 of *EPAS1*. In the single causal variant model, this SNP was the most significant one that correlated with a lower-than-expected Hb concentration for an altitude of 4200 m in another group of Tibetans on the Tibetan Plateau, among 31 significantly associated SNPs [12]. Lastly, SNP rs4953388 (G/A) is at position 46713201 on Chromosome 2 downstream of *EPAS1*. This SNP exhibited the most significant difference (*P* = 1.59×10^−9^) in allelic frequency between Tibetans (3,200–3,500 m in Yunnan Province, China) and non-Tibetan HapMap CHB subjects, out of 502,722 SNPs [12].

### Genotyping

Genomic DNA samples from Sherpa, non-Sherpa, J-HAPE-s, and J-HAPE-r subjects were extracted from venous blood leukocytes as described previously [8], [9], [24], [25]. The SNP Genotyping Assay Mix for rs13419896, rs4953354, and rs4953388 contained forward and reverse primers and FAM™ and VIC™ dye-minor groove binder-labeled probes (Applied Biosystems Inc., Tokyo, Japan). Allelic discrimination of the three SNPs was performed using a pre-designed 5′ nuclease assay (TaqMan® SNP Genotyping Assay according to manufacturer’s instruction with Applied Biosystems 7500 Fast Real-time PCR System (Applied Biosystems Inc., Foster City, CA, USA). Following thermal cycling, genotype data were acquired automatically and analyzed using sequence detection software (SDS v1.3.1, Applied Biosystems Inc.).

### Measurements of Serum EPO Levels in Sherpas at 3440 m and Non-Sherpas at 1300 m

Venous blood samples were taken from Sherpas at 3440 m and from non-Sherpa Nepalese at 1300 m. The sera were frozen at −20°C at the sampling locations. EPO measurements were conducted at Shinshu University, Japan, via radioimmunoassay using the Recombigen EPO RIA Kit (Mitsubishi Chemical Medience Corporation, Tokyo, Japan). The serum EPO levels in 72 Sherpas and 109 non-Sherpa Nepalese were available for the analysis.

### Statistical Analysis

Frequencies for genotypes, alleles, and haplotypes were expressed as decimals, and between-group comparisons were made with the Chi-square test. Hardy-Weinberg equilibrium for each SNP was confirmed with the Chi-square test in Sherpa, non-Sherpa, J-HAPE-s, and J-HAPE-r groups. Pair-wise genetic distances (*F_ST_)* between Sherpa, non-Sherpa, and J-HAPE-r subjects were measured following Weir and Cockerham [39]; comparing to the J-HAPE-s, the J-HAPE-r group was assumed to be more similar to the Sherpas in terms of hypoxia tolerance, and thus was selected for the calculation of *F_ST_*. LD blocks for the three tag SNPs were examined with Haploview 3.32 to calculate pair-wise LD measurements [40] such as D’, r^2^, and LOD. LOD provides a confidence measure of the D’ value; LOD>3 indicates that two loci are close to each other on the chromosome and therefore are likely to be inherited together [40]. The pair-wise LD measurements were then partitioned into block structures using common approaches to block definition [41]. Numerical values were presented as means ± standard deviation. Unpaired Student’s *t*-test was applied to compare EPO levels between Sherpa and non-Sherpa Nepalese subjects (two-tailed P 0.05 indicated statistical significance).
